# HIV-1 co-receptor usage:influence on mother-to-child transmission and pediatric infection

**DOI:** 10.1186/1479-5876-9-S1-S10

**Published:** 2011-01-27

**Authors:** Mariangela Cavarelli, Gabriella Scarlatti

**Affiliations:** 1 Unit of Viral Evolution and Transmission, Division of Immunology, Transplant and Infectious Diseases, San Raffaele Scientific Institute, Via Olgettina 58, 20132 Milan, Italy

## Abstract

Viral CCR5 usage is not a predictive marker of mother to child transmission (MTCT) of HIV-1. CXCR4-using viral variants are little represented in pregnant women, have an increased although not significant risk of transmission and can be eventually also detected in the neonates. Genetic polymorphisms are more frequently of relevance in the child than in the mother. However, specific tissues as the placenta or the intestine, which are involved in the prevalent routes of infection in MTCT, may play an important role of selective barriers.

The virus phenotype of the infected children, like that of adults, can evolve from R5 to CXCR4-using phenotype or remain R5 despite clinical progression to overt immune deficiency. The refined classification of R5 viruses into R5^narrow^ and R5^broad^ resolves the enigma of the R5 phenotype being associated with the state of immune deficiency. Studies are needed to address more in specific the relevance of these factors in HIV-1 MTCT and pediatric infection of non-B subtypes.

## Maternal viral co-receptor usage is not prognostic of transmission

The comparison of the co-receptor usage of viral variants obtained from transmitting and non-transmitting HIV-1 infected mothers demonstrated that most maternal viral isolates used CCR5 to infect target cells, alone or in association with other co-receptors, thus indicating that CCR5 usage is not a predictive marker of mother to child transmission (MTCT) of HIV-1 [1-4].

The high proportion of women carrying R5 virus prompted us to investigate if the intrinsic variability of these viruses may contribute to identifying a correlate of protection of MTCT. We made use of the newly introduced and refined R5 viral characterization, in which viruses are further classified in R5^broad^ or R5^narrow^ according to their capacity to use or not CCR5/CXCR4 chimeric receptors besides the wild-type CCR5 [5]. In particular, it was shown that during disease progression of infected adults R5 viruses evolved to multiple chimeric receptor usage, which in turn correlated with the CD4+ T cell decline in the patient. The use of chimeric receptors was interpreted as the evolution to an extended flexibility in the use of the CCR5, as R5^broad^ viruses have higher infectivity with the wild-type CCR5 than isolates with the R5^narrow^ phenotype.

Against our expectations we showed that mothers harbouring R5^broad^ viruses were not at a higher risk of transmission than those with R5^narrow^ viruses [6], thus again supporting that the R5 phenotype is not predictive of transmission. However, the maternal viral phenotype (either R5^narrow^ or R5^broad^) was generally preserved during transmission and predictive of the phenotype of the viral variant transmitted to the newborn.

Our original studies showed that the syncytium-inducing (SI), CXCR4-using viral variants were involved in MTCT of HIV-1 [4,7]. Indeed, HIV-1 infected mothers who harbor virus able to replicate in cell lines (rapid/high virus) and form syncytia in MT-2 cells had a higher although not significant risk of transmission than mothers with slow/low and non-SI viruses [4,7]. The number of mothers analyzed is however limited, and focused on subtype B HIV-1 infections.

A limited number of studies analyzed the role of the viral phenotype in MTCT within non-B HIV-1 subtypes. Indeed, subtype C followed by A, D, G and some circulating recombinant forms of HIV-1 are predominant in the world and specially in high endemic areas [8]. In pregnant women the major co-receptor for HIV-1 remains CCR5 also for viruses of subtypes A, C and G [9,10]. In addition isolates of these latter subtypes used frequently alternative chemokine receptors as for examples CXCR6 or CCR1, and rarely CXCR4 [1,11]. If these alternative chemokine receptor have a relevance is not yet clarified. It is of interest that CXCR6 is expressed on trophoblasts and may thus play a role for *in utero* transmission [12]. Further studies are needed to address if co-receptors others than CCR5 may have any relevance in HIV-1 MTCT of non-B subtypes.

## Selection or no selection: which virus is transmitted?

The very first studies comparing the genetic sequence of viruses from mother and child showed that the maternal viral population is more heterogeneous than that of the child [7,13-15]. If only a limited number of variants are originally transmitted and/or are initially replicating in the child is still a matter of discussion. On the one hand it was shown that a minor viral variant of the mother constituted the dominant variant in the child, on the other also a major maternal variant could be detected in the child [7,13,16-18]. If selective infection occurs, one could argue that an association between viral phenotype and transmission exist, however, all viral phenotypes, though to a different proportion, were detected in vertically infected children. On the other hand it was shown that selection may occur in relation to the different transmission routes, *in utero vs.* intra partum. Indeed, a major maternal virus variant as well as subtype C variants compared to A and D are associated with *in utero* transmission [14,15,19].

Indeed, R5 isolates are preferentially isolated from offspring [3,7,20-22], however, this could be a direct consequence of the higher frequency of mothers carrying R5 compared to X4 viruses. A more sophisticated analysis of the mother’s R5 viruses and of their child allowed us to pinpoint that the phenotype, either R5^narrow^ or R5^broad^, was usually maintained during the transmission event [6]. These data lend support to the lack of restriction in transmission of R5^broad^ viruses and favor the possibility that the maternal viral R5 phenotype is predictive of the transmitted variant.

On the contrary, the maternal R5X4 phenotype can be lost during transmission [6]. Mothers with an R5X4 virus transmitted virus with a whole array of phenotypes, i.e. R5^narrow^, R5^broad^ or R5X4. A definitive explanation for the inefficient transmission of X4 variants was not cleared yet. One possibility is that X4 variants are transmitted but rapidly deleted in the offspring, or that susceptibility of the child’s cells to infection by the mother’s isolate favors R5 variants, as discussed below. It is important to notice, that CXCR4 using variants were isolated from the children when the mother also carried such phenotype at delivery, indicating that transmission of X4 variants can occur with appropriate conditions. A recent study supports these data in non-B subtype virus infection [23]. Here the Authors demonstrate that in five Ugandan mother-child pairs X4 and R5X4 viruses are transmitted before, during or shortly after delivery, and thus, establish vertical transmission as an important source of CXCR4-using viruses in infants.

Another interesting observation comes from the study by Casper et al. [1], which shows that in HIV-1 infected children the emergence of the X4 phenotype during disease progression occurs when the mother carried an X4 virus. The same group was able to demonstrate that in these two children the X4 virus developed from their own R5 population, and not from a transmitted maternal X4 variant [24]. It is tempting to speculate that the transmitted virus has an intrinsic propensity to evolve to CXCR4 usage or that the similar genetic background of the mother and the child may favor such evolution.

## Viral phenotype correlates with pediatric disease progression

Children with perinatally acquired HIV-1 infection exhibit a widely variable clinical outcome: approximately one-forth of infants become symptomatic and develop AIDS within the first months of life, whereas the others remain asymptomatic or have only mild symptoms for several years [25,26]. A whole array of parameters, like the gestational period in which fetal infection occurs, and the child’s or the mother’s immunocompetent status, may influence infection outcome, and one of the crucial viral factors besides the viral load is the biological phenotype.

The virus phenotype of the infected children, like that of adults, can evolve from R5 to CXCR4-using phenotype or remain R5 despite clinical progression to overt immune deficiency [1,27-32]. Children progressing rapidly within a few years of age to disease may harbor R5 viruses but possibly have an elevated and fast increasing viral and proviral load associated [20,32].

X4 viruses are rarely isolated from neonates, and their predictive value of a rapid AIDS outcome showed discordant results [28,29,32-34]. Most of these reports unfortunately analyzed a limited number of children as to provide conclusive answers. Kopka et al. characterized 62 viral isolates of a cohort with an unusual high percentage (18%) of SI variants within the first 5 months of age, and showed that the presence of HIV-1 variants with rapid replication capability and/or an SI phenotype is indicative of a poor prognosis favoring CD4+ T cell depletion and rapid progression to AIDS [35]. On the contrary, in another study accurate sampling throughout the disease showed that CXCR4-using viruses possibly emerge in some children as a consequence of the severe immune deficiency [1]. In a recent study of a large cohort of 126 children and adolescent, included in the Hemophilia Growth and Development Study cohort, the baseline CXCR4 usage of their isolates predicted progression to clinical AIDS [36]. It can be argued that the two transmission routes, from the mother or through blood products, has different outcomes. However, if it is now well established knowledge that the CXCR4-using phenotype is associated with a severe state of immune deficiency, its predictive value is still controversial.

**Figure 1:**
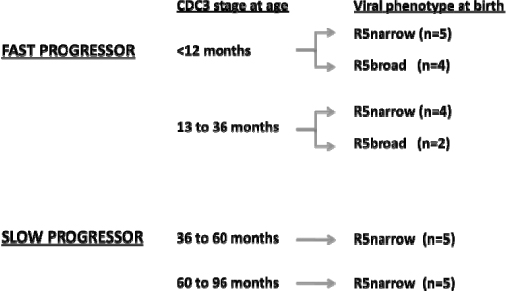
**The R5^broad^ phenotype is predictive of early immunological failure in children.** Categories are defined according to the Centers for Disease Controls [111]: CDC 3 = severe immune suppression. Narrow and broad refer to viruses with R5 phenotype detected at or close to birth. Viruses able to exclusively use wild type CCR5 receptor are defined narrow, whereas those using chimeric receptors besides the wild type CCR5 are defined broad. Statistical analysis was performed to detect the influence of the virus with R5broad phenotype on disease progression of the children; p = 0.0218 (Pearson’s chi Square).

Increasing evidences are emerging showing that the classical dichotomy of the viral phenotype into R5 and X4 is not sufficient to explain the large phenotypic variation of HIV-1 [5]. Further classification of R5 viruses into R5^narrow^ and R5^broad^ permitted to explain why some children progress more rapidly than others, despite the early presence of an R5 phenotype close to birth [6]. Our recent study performed on 28 infected newborns demonstrated that the presence of viruses with R5^broad^ phenotype close to birth was significantly associated with a fast progression to severe immunological failure within 3 years of age (Figure 1). Thus, infection in children established by R5^broad^ viral variants with an envelope conformation that allows for a more efficient CCR5 use, determine detrimental effects similar to those known for CXCR4 using viruses. The refined classification of R5 viruses into R5^narrow^ and R5^broad^ resolves the enigma of the R5 phenotype being associated with the state of immune deficiency. These data support the finding by Casper et al. [1], who suggested that the immunological deterioration in HIV-1 infected children precedes the viral phenotypic switch to CXCR4 usage. One could argue that pre-existing R5^broad^ viruses may have caused the worsening of the disease in the cohort analyzed by this study.

We documented also the phenotypic evolution from a wild-type R5 to broad chimeric receptor using viruses of HIV-1 infected children during disease progression [37] (and unpublished results), which is paralleled by an increase of CCL5/RANTES resistance of the virus *in vitro.* Our hypothesis is that virus variants may evolve towards a more efficient CCR5 usage and improved binding properties, possibly due to selection pressure exerted by the presence of chemokines abundant in HIV-1 infected persons [38,39]. Accordingly, R5^broad^ viruses may be considered escape variants as much as CXCR4 using viruses. It is desirable that this different sensitivity of R5^broad^ viruses to CCR5 ligands is further investigated since clinical trials introducing small CCR5 inhibitors for the treatment of pediatric HIV-1 infections are forthcoming.

## Relevance of co-receptor expression on HIV-1 cell targets

Since R5 viruses are predominant in MTCT of HIV-1, the interaction of R5 viruses with CD4+CCR5+ T cells may play an important role in the pathogenesis of pediatric AIDS. In neonates the memory CD4+ T cells (CD45RO+), which express high levels of CCR5, are 6 to 7 times less represented than the naïve CD4+ cells (CD45RA) [40]. The latter predominantly express CXCR4 [40-43] and were shown to be primarily infected by CXCR4 using viruses [44]. The paucity of memory CD4+ T cells in cord blood imply that CCR5-positive cells are relatively uncommon [42]. However, at later age, in infants and children, the majority of the HIV-1-infected CD4+ T cells in the blood display a memory (CD45RO+) phenotype [43]. The more efficient use of the wild-type CCR5 by R5^broad^ compared to R5^narrow^ viruses [5,37] supports the hypothesis that the R5^broad^ viruses may infect, in addition to memory cells, also CD4+ naïve cells despite the limited expression of the CCR5 molecule on their surface. Interestingly, although HIV-1 infected neonates in general had 10 to 100 fold higher number of infected CD4+ memory than naïve cells, those children who rapidly progressed towards disease had high proviral load in the CD4+ naïve cells [43,45]. It remains to be solved why CXCR4 using viruses are not maintained during transmission, despite the high prevalence of CXCR4+ naïve cells in neonates.

Besides CD4+ T lymphocytes HIV-1 infects also other cells of the immune systems, like macrophages and dendritic cells (DCs). The infection of monocytes/macrophages is of major importance since these cells act as reservoir of the virus. Cord blood derived monocyte/macrophages have an increased susceptibility to HIV-1 infection *in vitro* with R5 but not X4 virus compared to adult cells [46]. DCs, which are also abundant at mucosal sites, may have a relevant role being the intestine the preferential route of MTCT of HIV-1. Immature DCs do not express CXCR4 and allow infection through a CCR5-mediated process (*cis infection*), which would be in favor of a preferential R5 infection. However, DCs support transfer of virus, independent of co-receptor usage, *via* DC-SIGN (*trans infection*) [47].

All these studies underline the relevance of the expression of the different receptors for HIV-1 in cells derived from the peripheral blood, but little is known on cellular HIV targets in relevant tissues, as for example the gut or the placenta, of newborns and children (see chapter below).

## Genetic polymorphisms: determinants of expression of relevant receptors

Polymorphisms implicated in MTCT and pediatric disease progression are summarized in Table 1.

**Table 1:** Effect of genetic polymorphisms of HIV-1 receptors and ligands on HIV-1 mother–to-child transmission and pediatric disease progression.

Gene	Polymorphism	Influence on		Ref.
		MTCT risk	Disease progression	
CCR5	delta32	Decreased	Delayed	53,63
CCR5	59029A	Augmented	Accelerated	62
CCR2	64I	Contradicting results	Delayed	63-65
CX3CR1	I249	No effect	Accelerated	67
CD4	C868T	Augmented, when heterozygously expressed in the children	unknown	69
DC-SIGN	p-336C and p-201A	Augmented, when expressed in the children	unknown	70
DC-SIGN	exon 4 : R198Q, E214D, R221Q, and L242V	Augmented, when expressed in the children	unknown	70
DC-SIGNR	H1 and H3	Augmented	unknown	71
CCL3	Copy number variation	Augmented, when present in the children	unknown	72
SDF-1	3’UTR 801A	Augmented, when present in the mother	Accelerated	62, 73-76

Particularly interesting is a 32-nucleotide deletion (Δ32) of the CCR5 gene that renders the co-receptor non functional when homozygote or expressed to lower levels when heterozygote [48-51]. The incidence of the Δ32 CCR5 allele is high in Caucasian population, approximate 1% are homozygous and 20% heterozygous, but appears only sporadically in Asian and African populations [52]. A study on MTCT, described that the homozygous mutation confers resistance to infection [53]. However, in adults despite this genotype the protection is not absolute and some rare cases of HIV-1 infection were reported possibly due to transmission of CXCR4-using viruses. In MTCT there are no studies reported in this regard. The heterozygous form of this same 32-basepair deletion of the CCR5 gene, when detected in the HIV-1 infected mother does not decrease the risk of transmission [54-61]. It appears, however, to exert a protective effect against MTCT in those children exposed to a low maternal viral burden of an R5 virus isolate [2].

In sub-Saharan Africa, where MTCT remains an important route of infection but the Δ32 CCR5 deletion is rare, Singh et al. recently showed that the single amino acid substitution (G to A or C to T) at position 59029 or 59353 of the CCR5 promoter is significantly associated with risk of MTCT [62]. The association persists also when adjusted for CD4 counts and antiretroviral treatment of the mother. It is of utmost relevance to notice that the CCR5 mutation 59029 was differently represented in the three cohorts analyzed in this study, being the frequency highest in the South African children compared to the Malawian and Ugandan ones [62]. This underlines the variation depending on ethnicity and the importance of designing studies with appropriate control populations.

Several studies tried to find a correlation of the Δ32 CCR5 polymorphism with disease progression in infected children. The largest one, an international meta-analysis study, associated this genetic polymorphism with a decreased risk of death among Caucasian perinatally infected children, but only for the first year of life, whereas thereafter the effect was not any longer evident [63].

Chemokine receptors other than CCR5 were studied. A single nucleotide substitution of a valine residue for an isoleucine at position 65 in the CCR2 receptor (CCR2-64I) showed contradicting results when analyzed in relation to MTCT [64,65] but exerted a protective effect on disease progression in perinatally infected children [63]. Structural variants of the chemokine receptor CX3CR1, used by HIV-1 as co-receptor in the central nervous system [66], such as I249 and M280 affecting two amino acids (isoleucine-249 and methionine-280), were associated with rapid progression to AIDS in infected adults. However, in children only the CX3CR1-I249 genotype appears to be relevant for fast progression [67]. Furthermore, either these two chemokine receptors, CCR2 and CX3CR1, when studied specifically in sub-Saharan African populations do apparently not have a direct role in MTCT [62]. However, a Nairobi-based study suggests that the maternal CCR2-64I may partially protect against MTCT of HIV-1 by reducing baseline plasma HIV-1 viral load [68]

A polymorphism (C868T) of the CD4 gene, which is highly prevalent among Africans, plays a significant role in determining a two-fold increase of MTCT when heterozygously expressed in Kenyan children [69]. This same mutation had apparently no effect when expressed in the mother. If the change of the tertiary structure of CD4 induced by this single-nucleotide polymorphism may have a different effect on the association with the co-receptors and thus, selectively influence the binding and entry of R5 viruses compared to X4 viruses is not known.

DC-specific intracellular adhesion molecule-3-grabbing nonintegrin (DC-SIGN) and DC-SIGNR are C-type lectins that serve as HIV-1 receptor in addition to being cell adhesion and pathogen recognition receptors. DC-SIGN is expressed also on placental macrophages. Two promoter variants (p-336C and p-201A) as well as four protein modifying mutations in exon 4 (R198Q, E214D, R221Q, and L242V) of this gene, studied in Zimbabwean children born to HIV-1 positive mothers, are associated with intra partum, *in utero* and post partum HIV-1 transmission [70]. DC-SIGNR is expressed on the human placenta in the capillary endothelial cells. DC-SIGNR single nucleotide polymorphism, H1 and H3, studied in a large cohort of children born to HIV-1 infected mothers in Zimbabwe, is associated with increased infection during pregnancy and at birth [71]. Interestingly, this mutation produced lower levels of DC-SIGNR in placental tissue. The Authors speculate that this low production of DC-SIGNR may have implications on alternative infection mechanisms with loss of the protective role of the placental barrier, and possibly favor HIV-1 binding to CCR5, instead of DC-SIGNR, facilitating migration of maternal infected cells across the placental barrier.

The naturally occurring host genetic variants of the chemokine-chemokine receptor axis may have relevance in altering the receptor expression and the host immune response to HIV-1. One possible hypothesis is that the chemokine production of the fetus or child may affect transmission or disease progression. CCL3, 4 and 5 are the natural ligands of CCR5. CCL3 in humans is encoded by two functional genes CCL3 and CCL3-L1; their low copy number in the children but not in the mothers were shown to be associated with transmission of the virus [72]. A particular single nucleotide polymorphism in the CCL3 gene was encountered more frequently in infected children compared to exposed uninfected ones.

The homozygous mutation at position 881 of the 3’untranslated region of the SDF-1 gene (SDF-1 3’A), the ligand for the CXCR4, of the mother but not the infant was associated with MTCT [73]. Another study showed that the protective effect of the heterozygous form of the Δ 32 CCR5 is restricted by the SDF-1 genotype in HIV-1 infected children [74]. We showed that the presence of the SDF-1 3’A gene correlates with accelerated disease progression in HIV-1-infected children born to seropositive mothers but does not protect against MTCT of HIV-1 [75]. These data may indicated that the genetic polymorphism may allow for appearance of CXCR4-using virus variants during disease progression of the children, but that the same mutation does not have any effect on transmission given that most mothers carry CCR5 using viruses.

In studies conducted in Africa the mutated SDF-1 gene instead had no protective effect at all or some effect but only in postpartum transmission [62,73]. In addition, in a Kenyan study neither the Δ 32 CCR5 nor the SDF-1 3’A polymorphism were detected in a cohort of HIV-1 infected long term survivor and non-progressor children above 8 years of age [76]. This again underlines the need to identify different prognostic markers according to genetic background.

## Cellular targets in different routes of MTCT

Taken in consideration that transmission from the mother to the child occurs mainly through ingestion of infected fluids *in utero*, intra partum or via breast milk, or through the placenta it may very well be that HIV-1 utilizes receptor(s) other than CCR5 to infect cells present at the fetus/infant’s mucosal sites. Indeed alternative receptors used by HIV-1 were identified on mucosal epithelial cells. Intestinal enterocytes and M-cells were shown to selectively transcytoze virus through binding with galactosyl ceramide (GalCer) [77,78] or Fc receptor [79]. If enterocytes appear to favor *in vitro* the transport of R5 viral variants, M-cells instead favor X4 viruses. Rescigno et al. demonstrated *in vitro* and *in vivo* that DCs can penetrate through tight junctions of the intestinal enterocytes and favor transport of enterobacteria, invasive or not, from the intestinal lumen. Interestingly, CX3CR1 expressed on DCs was shown to be involved in the elongation of the DC’s cellular processes [80]. If HIV-1 utilizes a similar mechanism to invade the intestinal mucosa, and if it could select for a particular viral phenotype, remains to be determined.

Tonsils may also be a portal of entry for HIV-1 by ingestion. The tonsils mucosa contains M cells that lie above regions where DCs are juxtaposed with CD4+ lymphocytes [81,82]. In adults oral transmission occurs rarely [83,84] and HIV binding to tonsil epithelium exhibits limited progression to primary infection [85]. In the primate MTCT model, infection was shown to occur through the surface mucosa of the tonsil [86], where transmission of the virus may involve specialized M cells and DC capable of transporting HIV to the interior of the tonsil. Tonsil lymphoid cells have an increased susceptibility to infection with HIV-1 compared to PBMC, which may in part by ascribed to the increased expression of the viral co-receptor CXCR4 [87].

The other portal of entry specific for *in utero* MTCT of HIV-1 is the placenta possibly through a transannexial or transplacental passage. HIV-entry into trophoblastic cells has still to be elucidated, as primary trophoblastic cells express CXCR4 and CCR5 but not always CD4 on the surface [12]. HIV-1 was detected mostly in syncytiotrophoblasts, Hofbauer cells and placental macrophages of both early and late placentae [88-93], unless the mother underwent antiretroviral therapy from early on in pregnancy [94]. Trophoblastic cells derived from the outer layer of the healthy placenta or malignant trophoblastic cell lines are permissive to infection by laboratory strains of HIV-1 *in vitro *[95-97]. *In vitro*, HIV-1 can transcytose across a trophoblastic barrier or, alternatively, the infected monocytes and lymphocytes can rapidly fuse with trophoblastic cells independently from the viral chemokine-receptor usage [98]. DC-SIGN and ICAM-1 were implied to play a role in the passage of HIV from placental cells to Hofbauer cells or T-lymphocytes [99,100]. Furthermore, the maternal deciduals cells are more permissive to infection with R5 than X4 viruses. Moreover, those cells interact directly with the placental cells, which are permissive to cell-to-cell infection, especially during the first trimester of pregnancy. Despite this potential risk, *in utero* transmission is rare during this period, suggesting that a natural control of the virus may occur [101].

Human term placental cells express the recently discovered second receptor for CXCL12/SDF-1a CXCR7 (RDC1) [102,103], which is known to be a coreceptor for HIV-1, HIV-2 and SIV [104]. Somatic cells expressing CXCR7 show enhanced internalization of the chemokine suggesting that CXCR7 acts as a sink for SDF-1a [105]. If these mechanism may influence a preferential transmission of CCR5 using viruses at placental level remains to be elucidated.

Controversial data were reported regarding the expression levels of CCR5 on placental cells and the risk of vertical transmission. Indeed, up-regulation of CCR5 expression in the placenta was associated with MTCT of HIV-1 [106]. The same study revealed that HIV-1 infects primarily CXCR4-expressing cells in the placenta from non-transmitting women, but predominantly CCR5-expressing cells in those from transmitting women. However, a recent study did not find any correlation between the expression of CCR5 in human placenta and MTCT of HIV-1 in a cohort of mothers from Malawi [107]. Interestingly maternal malaria infection corresponded to a higher expression of CCR5 in the placenta, thus indicating that environmental factor are involved in the regulation of this molecule. In two African studies it was shown that Plasmodium falciparum infection profoundly modifies the placenta cytokine environment [108], and that placental malaria infection is associated with an increase in peripheral and placental HIV-1 load [109]. Indeed, the role of malaria infection in favoring MTCT of HIV-1 is still a matter of debate and needs further investigation. According to the latest estimates of WHO/UNAIDS in those areas where malaria is endemic were born the majority of the 430.000 newly HIV-1 infected babies [110].

## Conclusion

As to day viral phenotype was not identified as a predictive marker of MTCT of HIV-1. Genetic polymorphisms are more frequently of relevance in the child than in the mother. Mucosal tissues of the intestine or placenta appear to be involved in the selection of viral phenotypes, due to expression of specific receptors for HIV-1. The driving mechanisms need still further investigation.

The enigma of the R5 phenotype being associated with the state of immune deficiency has been solved with the refined viral characterization, in which viruses are further classified in R5^broad^ or R5^narrow^. Indeed, R5^broad^ viruses have detrimental effect as much as CXCR4-using viruses, and are predictive of fast disease progression in infected children.

## Competing interests

The authors declare that no competing interests exist.
